# A Second Soundly Sleeping Dragon: New Anatomical Details of the Chinese Troodontid *Mei long* with Implications for Phylogeny and Taphonomy

**DOI:** 10.1371/journal.pone.0045203

**Published:** 2012-09-27

**Authors:** Chunling Gao, Eric M. Morschhauser, David J. Varricchio, Jinyuan Liu, Bo Zhao

**Affiliations:** 1 Dalian Natural History Museum, Dalian, Liaoning, China; 2 Department of Earth and Environmental Sciences, University of Pennsylvania, Philadelphia, Pennsylvania, United States of America; 3 Earth Sciences Department, Montana State University, Bozeman, Montana, United States of America; Raymond M. Alf Museum of Paleontology, United States of America

## Abstract

A second nearly complete, articulated specimen of the basal troodontid *Mei long* (DNHM D2154) is reported from the Early Cretaceous (Hauterivian-Valanginian) lower Yixian Formation, Liaoning Province, China. New diagnostic features of *Mei long* are identified, including: a uniquely shaped maxilla, low with small, low maxillary fenestra; sacrum with an extremely wide caudal portion and elongate 4^th^ and 5^th^ sacral processes; and a large distal articular surface on the tibiotarsus which continues caudally on the tibia. A phylogenetic analysis including new data from the second specimen recovered *Mei* as a basal troodontid, in keeping with previous analyses. Although the skeleton exhibits several juvenile-like features including free cervical ribs, unfused frontals and nasals, and a short snouted skull, other attributes, full fusion of all neurocentral synostoses and the sacrum, and dense exteriors to cortical bone, suggest a small, mature individual. Microscopic examination of tibia and fibula histology confirms maturity and suggests an individual greater than two years old with slowed growth. Despite being one of the smallest dinosaurs, *Mei long* exhibits multi-year growth and cortical bone consisting largely of fibro-lamellar tissue marked by lines of arrested growth as in much larger and more basal theropods. This *Mei long* specimen lies in a similar but mirrored sleeping position to that of the holotype, strengthening the hypothesis that both specimens were preserved in a stereotypical life position. Like many Liaoning specimens, the new specimen also lacks extensive taphonomic and stratigraphic data, making further behavioral inference problematic.

## Introduction

The Jehol Group is composed of the stratigraphically conformable Yixian and Jiufotang Formations and is exposed in western portions of Liaoning Province, China. These formations are dominated by laminated and finely bedded siliciclastic sediments interspersed with extrusive basalts and tuffs [1]. They have produced a widely acclaimed fossilized fauna that includes a wide diversity of fish, invertebrates, plants [1], mammals [2], [3], diapsids [4], [5] including dinosaurs [6]–[8] with many specimens displaying exceptional soft-tissue and integument preservation [9]–[11]. These spectacular fossils are predominantly strongly compressed [12] and come from the siliciclastic sediments thought to have been deposited by a series of inland, freshwater lacustrine environments [1]. In the lower Yixian Formation, a fossiliferous tuff layer, the Lujiatun beds, crops out near Beipiao City [1]. The Lujiatun beds lack obvious bedding planes [1], and have been interpreted as volcaniclastic in origin, possibly representing volcanic mudflow events [13]. This results in beautiful three-dimensional preservation [13], [14]. However, see Meng et al. [15] for alternative scenarios. Several specimens have emerged from the lower beds with three-dimensional preservation provides the opportunity for behavioral inferences [13], [15], [16].

**Figure 1:**
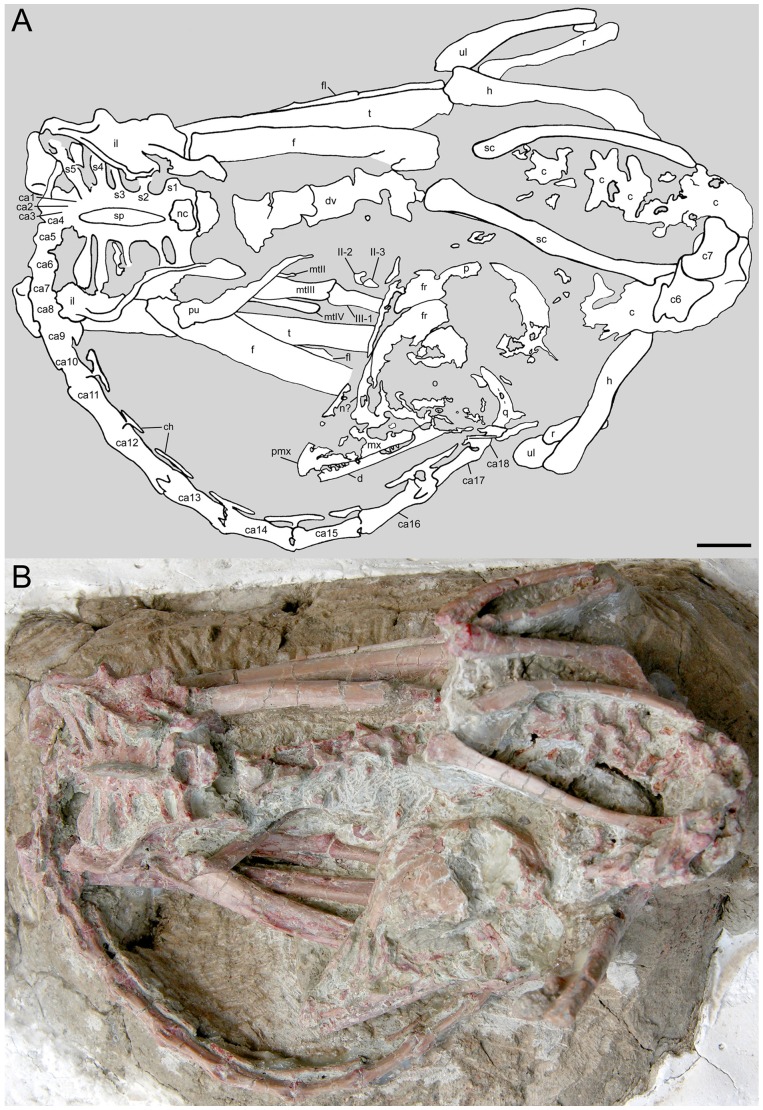
*Mei long* (DNHM D2514) in dorsal view. **A**, interpretive line drawing; **B**, photograph. **Abbreviations**: **aofe**, antorbital fenestra; **c**, cervical vertebra(e), unnumbered; **c6**, sixth cervical vertebra; **c7**, seventh cervical vertebra; **ca1–ca18**, caudal vertebrae (one through eighteen); **co**, coracoid; **d**, dentary; **dv**, dorsal vertebra(e); **f**, frontal; **fm**, femur; **fl**, fibula; **h**, humerus; **il**, ilium; **l**, lacrimal; **mt-II**, **mt-III**, second metatarsal, third metatarsal etc.; **mx**, maxilla; **mxfl?**, maxillary fenestra; **n?**, nasal; **nc**, neural canal; **ns**, neural spine; **o**, orbit; **p**, parietal; **pu**, pubis; **pmx**, premaxilla; **q**, quadrate; **r**, radius; **s1**, **s2**, etc., first sacral vertebra, second sacral vertebra etc.; **sa**, surangular; **sc**,scapula; **sp**, fused neural spine of sacrum; **t**, tibia; **u?**, possible manual ungual; **ul**, ulna; **II-2**, second phalanx of digit II of pes; **II-3**, third phalanx of digit II of pes; **III-1**, first phalanx of digit III of pes; **IV-1**–**IV-4**, first through fourth phalanges of digit IV. Note: Abbreviations follow the convention of Weishampel et al., 2004. Scale bar equals 1 cm.

One of these three-dimensionally preserved specimens is a small troodontid, named *Mei long* by Xu and Norell [16]. It was preserved in a position that closely matched the stereotypical sleeping posture of modern birds [16]. Here we report on a second specimen of *Mei long*, found in a nearly identical sleeping position (Figure 1), detailing its anatomy and aspects of its histology.

### Institutional Abbreviations

**DNHM**, Dalian Natural History Museum, Dalian, China; **IGM**, Institute of Geology, Mongolia, Ulaan Battar; **IVPP**, Institute of Vertebrate Paleontology and Paleoanthropology, Beijing, China; **MOR**, Museum of the Rockies, Bozeman, Montana, U.S.A.

## Materials and Methods

### Anatomical Terminology

For anatomical positioning, the terminology will follow the conventions of the Nomina Anatomica Avium [17] where feasible. In describing the orientation of the elements relative to each other as preserved, the terms “front” and “back” or “behind” are used to define directions along an axis roughly parallel to the dorsal series of the vertebral column, where moving from the sacrum towards the sharp curvature in the neck is defined as “forward” or the front. Consequently, moving from the neck towards the sacrum in any line roughly parallel to the dorsal vertebral column is defined as the rear, with terms indicating that direction including “rearward,” “back” or “behind.”

### Histology

To assess the ontogenetic age of DNHM D2154, we removed, molded and cast a section of the mid-diaphysis of the right tibia and fibula. A cast of the removed section was then inserted into the original specimen to retain the overall morphology. Cross-sections were prepared for each element using standard paleohistologic techniques [18]–[20], and examined with light microscopy. Descriptive terminology follows Francilion-Vieillot et al. [21], and age at death was assessed from annual lines of arrested growth (LAGs) [22].

### Phylogenetic Protocol

Our analysis used the matrix from Xu et al. [23] with coding modifications to *Mei* based on new information available from this specimen. The coding modifications can be found in the phylogenetic analysis section. Two troodontids published after Xu et al. [23] were added to the matrix. The coding for *Philovenator curriei* was obtained from Xu et al [24]. *Talos sampsoni* was conservatively coded from the literature [25]. This matrix was chosen because, while far more extensive matrices exist for understanding interrelationships across Coelurosauria (e.g. [26]) or the interrelationships of dromeosaurids [27], these matrices do not have as wide a sampling of published troodontid species as the Xu et al. [23] matrix.

The analysis was run in T.N.T. 1.1 [28], [29]. A seed of ten Wagner trees was used with 10000 replications of a traditional Tree-Bisection-Reconnection (TBR) search. A second round of TBR searching was conducted beginning with trees held in memory. There was no overflow with the second round of searching. Bremer support was calculated by sequentially increasing the suboptimal trees retained and examining the resulting strict consensus trees.

## Results

### Systematic Paleontology

Theropoda Marsh, 1881 [30].

Maniraptora Gauthier, 1986 [31].

Troodontidae Gilmore, 1924 [32] (Currie, 1987 [33]).

*Mei long* Xu and Norell, 2004 [16].

#### Holotype

IVPP V12733, a nearly complete, fully articulated skeleton.

#### Locality and horizon

Lujiatun, Shangyuan, Beipiao City, western Liaoning, China; lowest more fluvial and volcaniclastic beds of Yixian Formation, older than 128 and younger than 139 Ma [16].

#### Referred Specimen

DNHM D2154, a nearly complete, articulated skeleton including skull, complete cervical series, partial dorsal series, complete sacrum and ilium, a partial pubis, complete hind limbs, fairly complete caudal series and complete forelimbs, except for the furculum and much of the left manus. The missing forelimb elements may still be present in the matrix, but are not currently exposed.

#### Locality and horizon

Lujiatun beds, Kaoshangtun, Shangyuan, Beipiao City, western Liaoning, China.

#### Revised Diagnosis

*Mei long* is a troodontid with the following autapomorphies: Extremely large naris extending caudally over one-half of the maxillary tooth row [16]; closely packed middle maxillary teeth [16]; maxillary tooth row extending caudally to the level of the preorbital bar [16]; caudal portion of sacrum extremely wide with elongate 4^th^ and 5^th^ sacral processes; ilium strongly sigmoid in dorsal view, with a stronger lateral curve than in *Velociraptor* and *Anchiornis*; presence of a caudolateral flange on the midshaft of metatarsal IV [16]; the most proximal end of the pubic shaft significantly craniocaudally compressed relative to other troodontids [16].

Additionally, *Mei long* possesses the following combination of characters with a wider distribution: a bulbous and fully convex caudal portion of frontal and a short and steeply angled snout, features shared with *Anchiornis* and the Ukhaa Tolgod perinates (IGM 100/972 and 100/974) described as *Byronosaurus* [34], but perhaps representing a new taxon [35]; premaxilla with steeply inclined front and long maxillary process (shared with Ukhaa Tolgod perinates); maxilla, low with small, low maxillary fenestra and dorsoventrally short rostral process but thinner than in the Ukhaa Tolgod perinates; unserrated teeth as in *Anchiornis* and Ukhaa Tolgod perinates with straight distal margins as in Ukhaa Tolgod perinates, and *Sinovenator* but not *Anchiornis*; robust, sub-‘U’-shaped furculum more similar to those of oviraptorids; long ulna and radius approximately equal to or >90% of humeral length; metacarpal III longer than metacarpal II; unguals only moderately recurved compared to other maniraptorans; and distal articular surface of tibiotarsus large, continuing caudally on the tibia.

### Anatomical Description

The specimen (DNHM D2154) is encased in green mudstone with up to fine sand-size clasts. The individual is small, with a total length of approximately 325 mm and not more than 400 mm. This is some 130 to 200 mm shorter than the type specimen of *Mei long* (IVPP V12733). However, individual elements may provide a better estimate of the comparative size of these two specimens (Table S1). DNHM D2154 is consistently smaller than the original *Mei long* with individual linear measurements averaging 83% those of the holotype.

The skeleton is oriented dorsal side up from the base of the neck through the first caudals. The hind limbs are folded on either side of the torso (Figure 1). The forelimbs are both oriented with the elbows pointing up and slightly rearwards with the hands ventral, lying horizontally and directed rearwards. The only elements visible from the left manus are a large ungual, possibly phalanx I-2, and an elongate element. The latter cannot be clearly identified and may not be part of the manus. The right manus is better exposed, running rearward just beneath the skull. All elements are visible and in place except for metacarpal-I and possibly phalanx III-1. Although missing, an outline remains for metacarpal-I and an estimate can be made of its size and shape. No carpals are observable. The neck arches up and back so that the skull lies on the right side of the body, above the right knee and hand, behind the right elbow (Figure 1). The skull is resting on its right side and facing rearward. The tail also curls to the right and travels forward under the skull. Most of the tail is lying on its left side. Lying in this position the greatest front-rear and transverse dimensions of the specimen are 120 mm and 83 mm respectively. This posture is nearly identical to the avian sleeping posture of the type specimen of *Mei long* [16] and also bears some similarities to the curled pose seen in the type specimen of *Sinornithoides youngi*, IVPP V9612 [36]. Further basic measurements of the specimen are provided in Table S1.

### Skull

**Figure 2:**
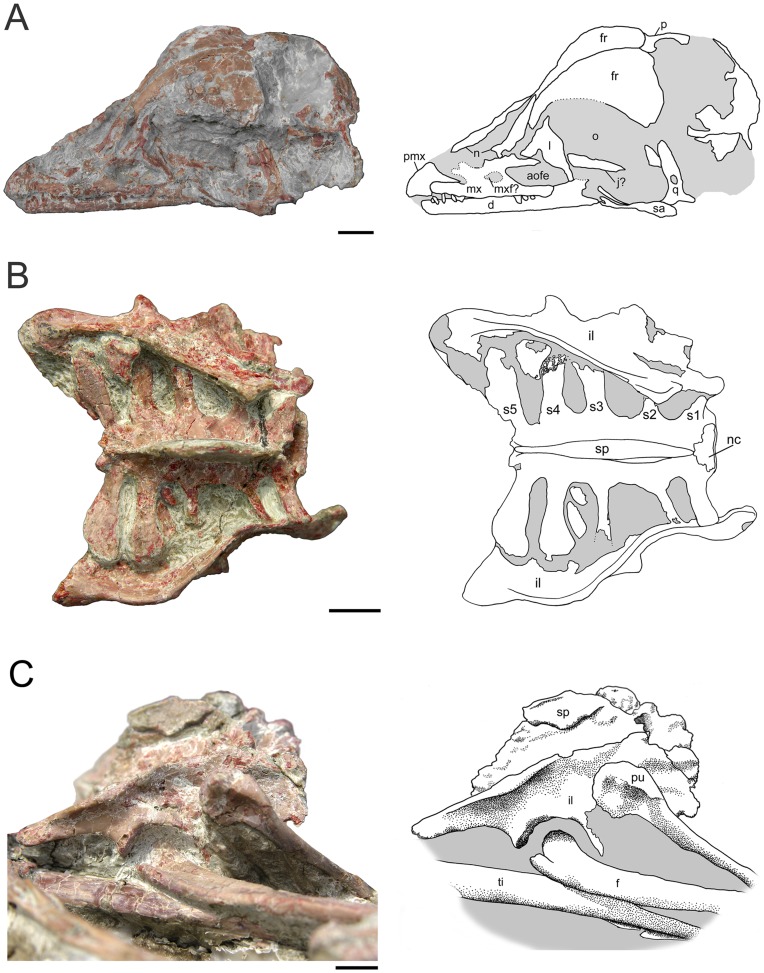
Photos and interpretive drawings for *Mei long*, DNHM D2514. **A.** Skull of *Mei long* in left lateral to left dorsolateral view. **B**, sacrum and ilia in dorsal view. **C**, pelvis in right lateral view. Abbreviations as in Figure 1. Scale bars equal 0.5 cm.

The skull (Figure 2A) is an estimated 5.0 cm long and has a steep anterior profile more reminiscent of *Archaeopteryx* [37]–[39] than other troodontids [40]–[42]. The skull to femur length ratio of *Mei long* is approximately 0.75, which is similar to the condition in *Sinusonasus magnodens* [16] and some other basal troodontids and dromeosaurs [36], [43], [44].

The relatively small premaxilla possesses a dorsal border that angles steeply back and a fairly stout caudal process that extends over the rostral most portion of the maxilla. It is impossible to determine whether this process excludes the maxilla from contributing to the nares or not. This subnarial process to the premaxilla is shared with *Sinusonasus* [44]; DNHM D2154 lacks the slit-like premaxillary fenestra seen in this taxon. The external naris cannot be delineated in DNHM D2154, however, it is elongate in the holotype. *Byronosaurus* is referred to as having a similarly extensive naris [34], but the left side of the skull appears to clearly show the naris ending at approximately 1/3 the total length of the toothrow [42]. The maxilla is 16.5 mm long and unusually low (approximately 1.4 mm deep), particularly over its rostral portion. The rostral process is lower relative to its length than any other troodontid. The maxillary fenestra is small and narrow dorsoventrally. The antorbital fenestra is larger than the maxillary fenestra and appears to be broadly triangular (Figure 2A). The interfenestral bar sits flush with the rim of the antorbital fossa in a condition reminiscent of *Byronosaurus jaffei* [42] and *Linhevenator* [23]. On the caudoventral portion of the bone a prominent slit-like groove occurs. A similar feature occurs in *Troodon formosus* and may accommodate the jugal.

The T-shaped lacrimal has a slightly concave dorsal surface. The full length of the rostral process is obscured by matrix. A small lateral extension sits just above the mediolaterally broad descending process. A matrix-filled recess occurs on the rostral face of this process, suggesting the possibility of a pneumatic fossa (recessus pneumaticus lacrimalis, Witmer [45]), as in most troodontids [34], [41], [46], or the opening of the lacrimal duct [42]. The nasals are unfused thin broad plates of bone. Both left and right frontals are nearly fully exposed in dorsal view (Figure 2A). They appear to be completely unfused along their entire lengths, as is the condition of the holotype. The caudal margin of each frontal is relatively straight, with a slight triangular enlargement at the midline where the frontals meet. The frontal is unusually broad and convex over its posterior half. This convexity continues to the lateral margin of the bone, unlike the condition seen in other troodontids (e.g. *Troodon*, *Zanabazar*) [47] and extends forward to the beginning of the rostral extensions of the frontal. In dorsal view, the frontal narrows rapidly at the level of the orbit. The lateral margin of the rostral portion of the frontal is dorsally upturned and thickened, presenting a slim vertical lateral face abutting the lacrimal. The rostral portion of each frontal is dorsally concave across a transverse transect. The frontal lamina on DNHM D2154 is similar to that reported for *Sinovenator* [14].

The caudal portion of the skull is not well preserved. Remnants of the parietal suggest it was broadly convex, confluent with the convexity of the frontals. A small portion of the supraoccipital is visible and suggests a broad central raised portion with shallow, caudal-facing depressions located laterally. The left quadrate is displaced rearwards revealing the moderately concave caudal aspect of the element. A sizable pneumatic foramen opens caudally at midheight (Figure 2B). The proximal portion tapers dorsally to a presumably narrow head, while the distal end flares for the articular surface. A slight ridge marks the caudomedial aspect of the element.

The dentary is long and narrows dorsoventrally toward the rostral end. It is 27.7 mm long along the ventral margin. At its caudal end, it is 3.6 mm tall, and it is about 1.5 mm tall at its rostral end. The dentary conforms to that of other troodontids in tapering rostrally and possessing a groove containing nutrient foramina along much of its length [33], [42], [46], [47], [48], [49]. The ramus of the dentary appears straight in ventral view, not curving towards the midline. This character is plesiomorphic for Troodontidae [42] being found in basal taxa [36], [42] and in the fragmentary *Urbacodon itemirensis* [50].

Middle and caudal maxillary teeth are laterally compressed and recurved. Teeth reach the greatest mesial-distal basal length (∼0.5 mm) near the mid length of the maxilla. Smaller teeth sit rostral and caudal. The teeth of the rostral dentary are narrow and tightly spaced. They appear similar to embryonic *Troodon* teeth [51] and are reminiscent of small, unidentified teeth of the Jurassic of Utah [52]. The first alveolus on the dentary is not exposed, so it cannot be determined if this specimen shares the small, rounded rostral dentary alveolus of *Archaeornithoides* [53]. DNHM D2154 appears to lack denticles on the teeth, as does *Byronosaurus* [42] and *Urbacodon* [50].

### Axial Skeleton

**Figure 3:**
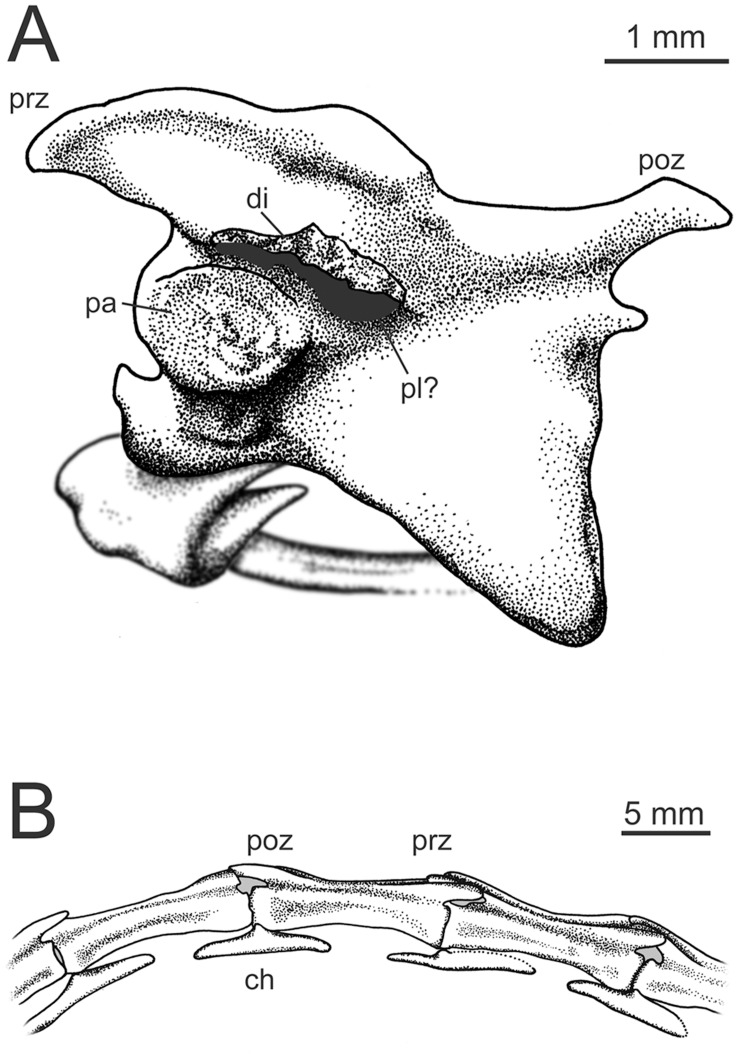
Details of the vertebrae of *Mei long*, DNHM D2514. **A**, Cervical no. 6 vertebra in left lateral view, scale bar equals 1 mm; **B**, the caudal end of ca 11, ca12–14 and the cranial end of ca15, in right lateral view (cranial to the right of the picture). Abbreviations as in Figure 1. Scale bar equals 5 mm.

Although portions of eight cervicals are visible, few provide good details (Figure 1). The following description is largely based upon cervicals 4, 5, and 6 (Figure 3A). The elongate, slightly keeled centra are far narrower than the broad, platform-like neural arches. Intercentrum articulations slope craniodorsally. The cranial articular surface is saddle-shaped and strongly concave in ventral view. The caudal articular surface appears to be broadly circular in outline and craniodorsally sloped. The parapophyses extend ventrolaterally from the sides of the centra near their cranial end (Figure 3A). Based on a few broken areas, the centra appear to be largely hollow with an undivided internal space.

At least two vertebrae show post-depositional fractures running through the centra, with some minor (∼5 mm) displacement between the respective ends. This is significant because there is no displacement between the centra and the neural arches. Further, these vertebrae lack obvious synostoses. Thus, arches and centra appear to be solidly fused. This is in contrast to the holotype, where faint synostoses are visible on the dorsal [16] and cervical vertebrae between the neural arches and the centra.

The neural arches are flat in horizontal view (Fig. 3A) and roughly rectangular in dorsal view. In ventral view, they extend laterally beyond the centrum. Overall form is very similar to that of other troodontids with relatively low neural spines (not well preserved in this specimen, clearer on the holotype); prezygapophyses that extend craniolaterally bearing broad, oval, cranioventrally angled articular surfaces; epipophyses small; and diapophyses located ventral to the cranial extensions for the prezygapophyses [54]. The diapophyses are also craniocaudally elongate. The low neural spines are located towards the caudal end of the arches. Two pairs of pleuorcoels exist on the neural arches. The first are oriented dorsolaterally and sit at mid-length on the dorsolateral aspect of the arch where the beginnings of pre- and postzygapophyses cross. This pair is apparently not found on a fragmentary troodontid specimen referred to *Saurornithoides mongoliensis* [55]. A second possible pleurocoel pair appears on cervical 6 (c6) as a matrix-filled pocket medial to the diapophysis on the ventral side (Figure 3A). The postzygapophyses are concave with the main portion of the articular surface oriented ventrally, but with a medioventral extension facing laterally. This extension is not as strongly angled relative to the postzygapophyseal articulation as in the typical hyposphene condition (e.g. *Sinraptor dongi* [56]). Concave postzygapophyses occur in *Troodon* and elsewhere. The cervical ribs appear to be unfused (Fig. 3A).

Although portions of nearly all the dorsal vertebrae are visible, few provide good details (Figure 1B). The visible portions include partial dorsal views of the neural arches of dorsals (D) 1–5 and lateral views of D8–13, but the latter are damaged.

The dorsal view of D2 exhibits a low neural spine that is craniocaudally short and sits near the caudal margin of the arch. The arch is wider than long. The long transverse process is caudolaterally swept. The articular facets on the prezygapophyses are large and face dorsomedially. The postzygapophyses sit closer to the midline than the prezygapophyses and project caudolaterally from near the base of the neural spine. The width of D2 across the transverse process is 10.5 mm and its length is 5.7 mm from the pre- to the postzygapophyses. The neural spine of D3 is taller and craniocaudally longer than that of D2, and the transverse processes project laterally, lacking the caudal sweep seen in the latter.

The centra of D8–13 are spool-shaped, with somewhat concave sides that become increasingly so as one moves toward the sacrum. No pleurocoels are evident. The neural spines of these vertebrae are rectangular in outline. The most complete neural spine (from D8) measures 4.2 mm above the rest of the neural arch. On D11 the craniocaudal length of the neural spine is about 64% the length of the centrum. Although few troodontid dorsal vertebrae are known, those figured for *Sinovenator* [14] and *Anchiornis* ([57] Figure S3C) are strongly similar to *Mei long*.

The sacrum is visible in dorsal view, where it is both well-preserved and well-exposed (Figure 2B). It has a total length of 22.2 mm. The sacral count of 5 matches that of the holotype of *Mei* [16], *Sinusonasus* [44], and *Sinovenator* [14], but is lower than that of *Saurornithoides* [48] and *Troodon*. The neural canal is particularly broad at S1, nearly 5.5 mm across, approaching the width of the D12 centrum (6.4 mm). Overall, the sacrum widens caudally with a maximum width at sacral processes 4 and 5 (Fig. 2B). This is in contrast to the significantly more modest caudal widening known for *Sinovenator* and basal dromeosaurs [14]. The width of the sacral centra in these other taxa and DNHM D2514 is greatest across the third centrum. The preserved portions of the sacral processes of the holotype are consistent with the pattern seen in DNHM D2154. The sacrum appears to have a slight dorsal arc to it (Figure 2C), although this may be accentuated by slight dorsal tilting of the cranial end. The neural arches of the five sacrals appear fully fused, forming a broad, slightly irregular platform (Figure 2B) at the base of the fused, plate-like neural spine (Figure 2C). This condition is also seen in *Zanabazar* [47], [48].

Five sacral processes extend from each side of the sacrum (Figure 2B). All of the sacral processes have a slight distal upturn. The two cranial most pairs extend slightly craniolaterally and are attached cranially to the lateral surface of their respective vertebrae. They have ovate cross-sections. The third, slightly longer pair attaches to S3 at mid length on S3 and extends directly laterally. The third pair also has a ventral process that extends laterally beyond the dorsal portion. The cross-section of this pair of processes is hourglass- or dumbbell-shaped. The first three pairs of sacral processes maintain fairly constant widths throughout their length.

The caudal-most two pairs of sacral processes are significantly longer and broader in dorsal view (Figure 2B). The fourth sacral process begins to expand at mid-length, terminating in a roundish form. Throughout its length, the fourth process has a T-shaped cross-section resulting from a deep, cranioventral process. The fifth and most caudal process is more fan-shaped in dorsal view, expanding in width beginning near its base. This process angles slightly caudally and appears to also have a T-shaped cross-section. Narrowly spaced, postzygapophyses extend from the caudal end of the sacrum.

The caudal series changes dramatically throughout the first nine vertebrae (Figure 1), whereas caudals (ca)10–18 retain a fairly uniform morphology (Figure 3B). The first few caudals are craniocaudally short, with modest neural spines. The transverse processes project laterally in a frontal plane. From ca3 or ca4 through ca8 the neural spines and transverse processes diminish in size, pre- and postzygapophyses shift closer to the midline of the centra and the centra elongate slightly. By ca7, zygapophyseal surfaces are close to the midline and nearly vertical in orientation. A craniocaudal sulcus develops dorsally in this region, so that in ca7, ca8 and ca9, the base of the neural spine sits in a shallow depression.

Vertebrae caudal to ca9 exhibit a fairly consistent shape (Figure 3B). The centra are elongate (the longest centra in the tail being ca11 and ca13) with flat to slightly concave sides and a slight lateral ridge running craniocaudally at the junction of the centrum and neural arch. All the caudal vertebrae in this section lack neural spines and transverse processes, but they do possess a dorsal sulcus running craniocaudally from the pre- to the postzygapophyses. This sulcus is also seen on the distal caudals of *Saurornithoides mongoliensis* [55], *Byronosaurus* [58], *Sinornithoides* [36], [46], *Sinusonasus* [44], and *Troodon formosus*. The sulcus on ca10–ca13 shallows at mid-length, partially interrupting the groove. Pre- and postzygapophyses are elongate and low, overlapping the centra of preceding and succeeding vertebrae (Figure 3B). The slightly longer prezygapophyses diverge slightly laterally. The postzygapophyses, running nearly parallel to the midline, then fit into the slight pocket created by the diverging prezygapophyses. Although the last neural spine and transverse processes occur on ca9 of the visible 18, this represents a small portion of the overall tail length. The total length of caudal vertebrae 1–17 is roughly 130 mm, but the first nine vertebrae make up only 43 mm, or 33%, of the preserved tail length.

At least eleven chevrons are exposed on the specimen. The chevrons following ca5, ca7 and ca8 are poorly exposed, and a single disarticulated chevron may follow ca4. The putative chevron following ca4 is an elongate chevron with the long axis perpendicular to the articular surface. It is tall and narrow, with a maximum dorsoventral dimension much greater than its maximum craniocaudal dimension. The articulation consists of two conjoined nearly circular and slightly convex articular facets that face slightly mediodorsally. The overall outline of the articulation is barbell-shaped. This is in contrast to the chevrons of *Saurornithoides*, whose articulations do not meet at the midline [48]. The somewhat laterally compressed shaft bears two cranial and two caudal projections slightly distal to the articular facet. The cranial projections extend craniodorsally, reaching or slightly surpassing the level of the articulation. Overall these proportions are very similar to cranial chevrons of *Troodon formosus*.

Chevrons of both ca7 and ca8 have a much broader-based appearance, with a dorsoventral height only slightly greater than their craniocaudal length. They are also significantly craniocaudally broader in the shaft near the proximal articulation than chevron ca4. Oddly, both shafts appear to angle cranioventrally rather than caudoventrally as would be expected. The hatchet-shaped morphology of a mid-caudal chevron for *Anchiornis* ([57], Figure S3f) is not represented in this specimen of *Mei*. It is possible that the chevrons for ca7 and ca8 simply represent hatchet-shaped chevrons whose caudal processes have broken.

The chevrons following vertebrae ca9–ca16 have a flat morphology, having a maximum craniocaudal dimension that vastly exceeds their maximum dorsoventral dimension by many times (Figure 3B). The articular facets, located at mid-length, represent the dorsal-most point of the element. Dorsoventrally flattened extensions run both cranially and caudally from the articular facets. These bifurcate near their ends and may have overlapped with succeeding chevrons. The ventral aspect of these chevrons is flat.

Many of the features of the caudal series of *Mei long* (DNHM D2154) compare favorably to the well-preserved tail in *Sinornithoides* [36], [48]. The neural spines in both individuals are absent by ca10. The transverse processes become faint ridges after ca9. The chevrons become flat with a similar morphology beginning with ca8 [36] or ca9 [48]. The transition point in the tail is between ca9 and ca10. The centrum length jumps sharply at this transition point, and the longest caudal centrum is ca13.

None of the dorsal ribs of DNHM D2514 are preserved in sufficient detail to warrant description.

### Forelimb and Pectoral Girdle

**Figure 4:**
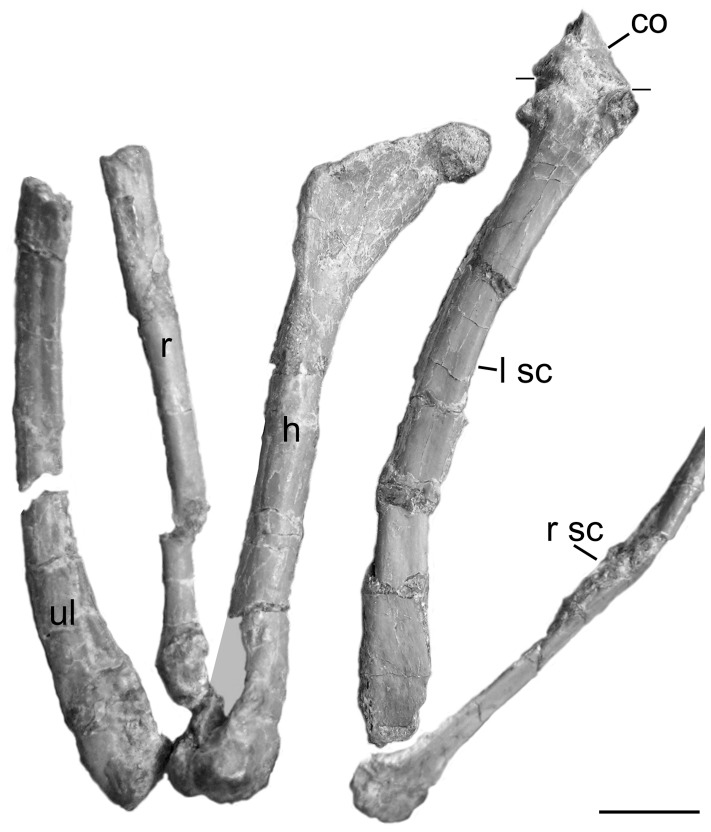
Detail of left forelimb of *Mei long* DNHM D2514 in articulation. Photograph showing scapula and coracoid (caudolateral view), humerus (mediolateral view), and partial ulna and radius (lateral view), as well as a portion of the right scapula (dorsal view). Lines on the left scapula demarcate border with the coracoid. **Abbreviations**: **co**, coracoid; **h**, humerus; **lsc**, left scapula; **r**, radius; **rsc**, right scapula; **ul**, ulna. Scale bar equals 0.5 cm. [planned for 2/3 page width].

The shoulder girdle is preserved in situ (Figure 4), with the scapula extending caudodorsally from the glenohumeral joint and the coracoids extending towards the midline in a transverse plane. The left and right humeri project caudodorsally and laterodorsally, respectively. The elbow is sharply bent so that the ulna and radius on both sides extend forward and down. Both mani, folded at a 90° angle to the ulna/radius, extend rearward.

The elongate scapular blades run near parallel to the dorsal vertebral column (Figure 1). The scapula is much shorter relative to the femur in *Mei* than in *Linhevenator* [23]. The scapular blade is mediolaterally thin and dorsoventrally narrow. It shows a slight but distinct expansion at its caudal end. The cranial end shows no dorsal expansion for a pronounced acromion as in most other theropods. This is similar to the condition seen in *Troodon formosus*. There is also a moderate cranial extension along the line of the dorsal edge of the blade. Just ventral to this expansion is a lateral concavity. The glenoid appears to open ventrally, and the position of the left humerus is consistent with this interpretation (Figure 4). This is in contrast to the early troodontids, *Sinornithoides* and *Sinovenator,* whose glenoid cavities face mostly caudally [46] and laterally [14] respectively. The glenoid opens ventrally in the derived troodontid *Linhevenator* [23]. The scapula and coracoid on the left side may be fused. When viewed dorsally, the coracoid meets the scapula at an angle of 90° to the long axis of the scapular blade. The coracoid also bears a prominent coracoid tubercle near its ventrolateral border. A slender element, seen only in cross section, near the acromion of the scapula appears to be a clavicle, but must be a rib, as the furcula of *Mei* is thin, but broad [16]. In its breadth, the furcula of *Mei* is more like that of oviraptorids than dromeosaurids [59].

The humerus has a narrow proximal end terminating in a rounded articulation. The slender shaft arcs slightly craniolaterally resulting in a concave medial edge when viewed distally and a concave caudal edge when viewed laterally. The triangular deltopectoral crest projects craniolaterally. Caudally, a rounded ridge forms its lateral edge, demarking a shallow caudal depression. Overall the deltopectoral crest in *Mei* is relatively short and extends 22% of the length of the humerus (Figure 4). The deltopectoral crests of other basal troodontids, such as *Sinornithoides* (19 mm and 23% of the humeral length), are similarly short [36] in contrast to the proportionally long deltopectoral crest seen in *Linhevenator* [23]. The shaft of the humerus of DNHM D2154 is ∼2.5 mm in diameter at midshaft, but expands mediolaterally at the distal end. The humerus is much more slender compared to its length in *Mei* than in *Linhevenator* [23]. The humerus is much longer relative to the femur, being 55% of the femur length, than is seen in *Linhevenator* [23]. A well-developed, groove-like olecranon fossa separates the two distal condyles caudally. The medial condyle has a simple rounded form, while the lateral condyle is slightly divided. The coronoid fossa is a shallow, laterally facing circular depression that sits just proximal to the latter. A small rugose area occupies the lateral edge of the humerus just proximal to this concavity. The geometry of the right elbow does not match that of the left. Furthermore, the distal end of the humerus and proximal ends of the ulna and radius are paler, with a more fibrous bone texture. These features suggest that the right elbow was reconstructed. All descriptions in the preceding paragraph come from the left forelimb.

The distal most portions of the left ulna and radius are lost, but comparable portions are preserved on the right side. The ulna has a roughly triangular proximal articulation and an expanded distal end (Figure 4). This distal articulation remains laterally compressed but is strongly rounded in lateral view. The shaft is laterally compressed, yet nonetheless bowed as in other maniraptorans [31] and compares well to the condition in *Microraptor* (Hwang et al, [60] Fig. 20). This is in contrast to *Anchiornis* and *Talos*, which possesses a straight ulna [57], [25]. The ulna is at least 86% the length of the humerus and more likely greater than 90% of that length (due to estimates of humerus length). In *Sinornithoides*, the ulna is 80% the length of the humerus [46].

Neither radius is well preserved. The element has a straight, slender shaft with a circular cross-section (Figure 4). The midshaft diameter measures 1.4 to 1.5 mm. The distal radius is lunate, as in *Sinornithoides* [36] and *Troodon formosus*.

**Figure 5:**
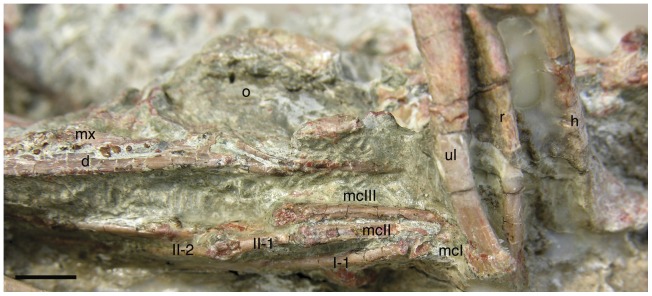
Details of the right manus in lateral view and skull in left lateroventral view. Manus shows the unusual features of reduced divergence in digit I and metacarpal III longer than metacarpal II. **Abbreviations: I-1**, **II-1**, and **II-2**, corresponding manus digit and phalanx; **mcI**, **mcII**, and **mcIII**, metacarpals for digits I–III; and other abbreviations as in Figure 1. Scale bar equals 0.5 cm.

Due to encasing matrix, carpals are not observable in this specimen. The metacarpals are of distinct proportions (Figure 5). Metacarpal-I appears to have been quite short, with a ginglymoid distal articulation. Total length likely measured about 3.7 mm and about 28% the length of metacarpal-II. As preserved, the orientation of digit-I suggests that neither metacarpal-I nor its digit diverged from digit-II. Thus metacarpal-I many have had a straight rather than divergent morphology, unlike most theropods [54], [61]–[67]. The elongate and slender metacarpal-II ends with a ginglymoid distal articulation. Metacarpal III is the most unusual, in being nearly as mediolaterally wide (at 0.9 mm) as metacarpal II (at 1.1 mm) and longer. Metacarpal III is 14.3 mm long relative to metacarpal II at 13 mm (Figure 5). These lengths are estimations, as the proximal end of the metacarpals remain obscured by matrix and the distal ulna. Bone is present lateral to metacarpal II to its proximal end, but whether this is the broken proximal end of metacarpal III or a distal carpal is unclear. In both regards this is unusual for theropods, which typically have a very slender metacarpal III shorter or subequal in length to metacarpal II [63], [68]–[70]. A more robust metacarpal III is seen in *Xiaotingia* [71]. A slightly longer metacarpal III is seen in *Scansoriopteryx heilmanni* [72], *Epidendrosaurus ningchengensis* [73], and the ornithomimids *Harpymimus okladnikovi* and *Anserimimus planinychus* [74]. It is also possible that metacarpal III is simply distally displaced, as is seen in many deinonychosaur specimens [43]. The shaft of metacarpal III bows out laterally (Figure 5), and it has a convex extensor surface; the shaft is deeper than broad. Metacarpal III has a rounded, apparently undivided distal articulation.

The phalanges of the manus are all narrow and elongate with ginglymoid articulations (Figure 5). The longest phalanx is I-1 at 14 mm, followed by II-2 (∼13 mm) and III-3 at 10.7 mm. The long penultimate phalanges suggest a well-developed grasping action to the manus [65]. The narrow unguals are typical in form, but only moderately recurved, with pronounced blood grooves and robust flexor tubercles. The ungual of digit I is the largest at 7.3 mm, followed by that of digit II. The unguals lack the dorsoproximal ‘lip’ seen in *Chirostenotes pergracilis* and some other small theropods [75].

### Hind Limb and Pelvic Girdle

**Figure 6:**
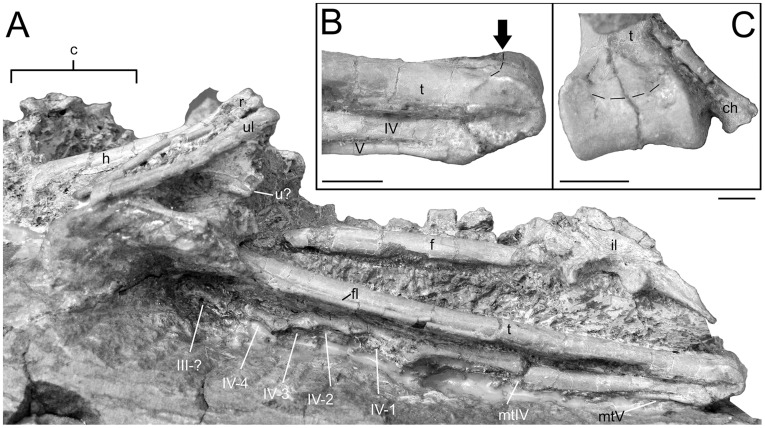
Hindlimb details of *Mei long* DNHM D2514. **A**, articulated left hindlimb in lateral view; **B**, distal tibia and metatarsals IV and V of left hindlimb. Arrow and dashed line indicate caudodorsal edge of articular surface; **C**, distal end of tibia in caudodistal view. Dashed line as in **B**. Abbreviations as in Figure 1. Scale bars equal 0.5 cm.

Both ilia are exposed in lateral and dorsal views (Figure 2B, C). The left ilium is slightly crushed, and the dorsal portion of the blade is eroded (Figure 6A). Only the proximal portions of the pubes are visible (Figure 2C). Neither ischium is visible.

The ilium has a total length of 34.4 mm, which is relatively small (54%) compared to the total length of the femur (64.4 mm). A relatively small ilium is a common paravian feature [14], [57]. The craniocaudally long acetabulum (9.8 mm) is centrally-placed relative to the iliac blade. The articular surface is strongly arched and relatively wide (3.4 mm at mid-length). The relatively small pubic peduncle marks its cranial limit. The supraacetabular rim overhangs the anterior two-thirds of the acetabulum laterally. The caudal most portion of the acetabular surface expands to a sub-circular, craniolaterally everted face. The pubic peduncle is longer and deeper than the ischial peduncle, as seen in many maniraptorans [46], [76], [77]. The ischial peduncle lacks a distinct articular facet for the ischium.

The iliac blade appears both low and short relative to the length of the acetabulum. Although the iliac blade is equally long, both cranial and caudal to the acetabulum, the cranial portion is significantly deeper dorsoventrally (Figure 2C). Cranial to the pubic pedicel, the blade consists of a vertically oriented, flat to slightly laterally concave lamina with straight cranial and dorsal margins. The ventral border is emarginated. Caudal to the pubic pedicel, the blade changes drastically. The upper edge of the blade angles caudoventrally and, caudal to the ischial pedicel, the blade tapers sharply. The upper edge also flares laterally, slightly overhanging the more ventral portions. Consequently, this dorsoventrally narrowing portion of the iliac blade has a concave lateral aspect.

As the blade narrows dorsoventrally, it also flares laterally, accommodating the much longer 4^th^ and 5^th^ sacral processes (Figure 2B). Thus the caudal half of the pelvis is significantly wider than the cranial. Though caudal widening of the ilium is known for deinonychosaurs [57], [60], [69], such marked sinuous structure of the ilia has not been demonstrated in this group (though it may be present in *Microraptor*, the crushing of the specimens prevents evaluation). The preserved sacrum of *Anchiornis* [57] does not support such dramatic caudal flaring. Dramatic caudal flaring of the ilium is a derived characteristic of therizinosaurs (e.g. *Segnosaurus* [78], *Nothronychus* [79]), but is ambiguous in the basal therizinosauroid *Falcarius* [76]. Despite having similar degrees of caudal flaring to *Mei*, the dorsal margins of these thereizinosauroids are not as markedly sigmoid, due to the greater lateral flaring of the preacetabular ilium [78]. The caudal termination of the ilium of *Mei* is relatively flat ventrally (Fig. 2C) but rounded in dorsal view (Fig. 2B). The depth of the cranial portion of the blade is 8.3 mm. The depth of the pelvis at the pubic pedicel is 12.3 mm and the depth of the pelvis at the ischial pedicel is 7.7 mm. The interior length of the acetabulum is 7.8 mm. The sacrum obscures much of the medial aspect of the ilium. The caudal portion of the ilium is inclined ventromedially from just behind the 3^rd^ sacral process to the caudal limit of the bone. The postacetabular blade of the ilium is longer than the preacetabular blade. This makes the ilium dolichoiliac. The preacetabular blade of the ilium is 11.2 mm long. The postacetabular blade of the ilium is 12.5 mm long.

The proximal ends of both pubes are preserved angling cranioventrally and towards the midline (Figure 2C), suggesting that the pelvis of DNHM D2154 has a forward-facing pubis. This is in contrast to the basal troodontid *Sinovenator*, which has a retroverted pubis [14]. However, other basal troodontids (*Sinusonasus*, *Sinornithoides*) also have a cranioventrally directed pubis [36], [43]. The pubis has a vertically oriented contact for the ischium, a fairly broad (3.6 mm wide) slightly laterally facing contribution to the acetabulum and a small, flat articular facet for the ilium in a dorsal plane. A distinct depression marks the lateral side of the proximal end, just before it tapers to the narrow shaft. The proximal end of the pubis is similarly concave in medial aspect.

Neither the left nor the right femur is well exposed, with the pelvic bones and matrix obscuring portions on both sides (Figure 2C, 6A). In contrast to the gently craniocaudally arched femur of the type, the shaft in this specimen appears straight (Figure 6A). This may reflect lithostatic crushing in the referred specimen. The total femur length is estimated at 65 mm (80% the length of the type specimen). This length is small, even for the smallest troodontids: that of *Anchiornis* measures 66.2 mm [57]; *Sinusonasus*, 141 mm [43]; and *Sinornithoides,* 140 mm [36]. The left femur is undistorted and largely circular in cross-section, the right femur is craniocaudally flattened. A ridge extends distally off the lesser trochanter and marks the craniolateral margin of the proximal shaft.

As with the femur, much of the tibia is obscured (Figure 6A). The estimated total length of the tibia and the astragalus is 86.1 mm. The shaft of the tibia is straight. It is subtriangular in cross-section proximally, becoming a flattened oval distally. The body of the astragalus appears to be fused to the tibia forming a tibiotarsus similar to that seen in *Sinusonasus* [43]. However, a broken corner suggests that the ascending process was not fused. There is no indication of a free calcaneum, so that element is either fully fused to the astragalus or not ossified in this taxon. The calcaneum is completely fused to the astragalus in *Sinornithoides* [36] and large individuals of *Troodon formosus* [25]. In contrast, the calcaneum and astragalus are unfused in *Talos* [25]. The articular surface is a smooth roller-like structure with raised lips both medially and laterally (Fig. 6B, C). These ridges have their beginnings on the distal portion of the tibial shaft. The articular surface sweeps through a large angle of around 270°, continuing on to the posterior surface of the astragalus and tibia. The tibia and astragalus of *Zanabazar junior* match the condition seen in DNHM D2154, except for lacking the articular surface extending onto the caudal face [47]. A juvenile specimen of *Philovenator curriei* possesses an astragalus that covers the entire distal end of the tibia, but does not extend on to the caudal surface of the tibia [80].

The fibula is a very slender element, whose proximal end remains buried in matrix. It bears a small, but distinct iliofibularis tubercle about 20% down its length from the proximal end. The tubercle measures about 2.5 mm in length and faces craniolaterally. This is similar to the laterally projecting process of *Sinornithoides* [46]. The remainder of the fibular shaft is slender, extends onto the cranial aspect of the tibia, and appears to reach the proximal tarsals. The shaft diameter at mid-length is only 0.7 mm and significantly narrower than that of the tibia. As in most Asian troodontids, the fibula and the tibia are not fused to each other [43], [46], [47], [81].

The pes is only partially exposed. Visible portions of the right pes include distal metatarsal III, distal metatarsal IV, phalanx II-2, unguals II-3, and III-1 (Figure 1). Visible portions of the left pes include metatarsal IV, metatarsal V, phalanx III-3, phalanges IV-1, IV-2, IV-3 and IV-4 (Fig. 6A).

The metatarsals are very long relative to the femur, as is seen in *Sinornithoides* [36] and in contrast to *Linhevenator* [23]. Metatarsal III is observable in cranial view only. The shaft of metatarsal III appears wedged between those of II and IV distally. The flat to slightly concave cranial aspect of the shaft expands distally, reaching a maximum width of about 3.5 mm at a point 11 mm proximal to the distal end. The shaft then narrows, particularly emarginating on the medial side where it creates a gap between metatarsals II and III, before it expands slightly at the distal articular surface. The distal articulation is smoothly rounded and undivided. The total length of metatarsal III and the overall metatarsus is estimated to be 49 mm. Metatarsal II appears shorter than both III and IV. Metatarsal IV is elongate but slightly shorter than metatarsal III, mediolaterally compressed with a narrow distal condyle and a lateral flange extending caudally from its shaft. A small collateral ligament pit sits on the lateral surface just proximal to the distal articulation. Metatarsal IV appears more slender than metatarsal III. This is in contrast to the relatively robust metatarsal IV found in the more derived troodontids *Sinusonasus* [44], *Tochisaurus nemgetensis* [82], *Borogovia gracilis* [81], *Sinornithoides* [36], [46], the unidentified troodontid IGM 100/44 (elsewhere SPS 100/44) [54], *Saurornithoides mongoliensis* [55], *Talos* [25] and *Troodon formosus* [83], [84]. The other Liaoning troodontid, *Sinovenator*, appears to have a third condition where metatarsal IV is significantly more robust than metatarsal III, but only slightly more robust than metatarsal II [14]. Metatarsal V is a narrow splint-like bone located at the caudolateral corner of metatarsal IV [14], [46]. It appears to extend and re-contact metatarsal IV just prior to the beginning of the posterior flange of the latter’s shaft, as in the juvenile troodontid described by Currie and Peng [80].

Just enough of digit II is visible to indicate a retracted phalanx II-2 supporting the ungual. Phalanx III-1 appears to have an undivided proximal articulation but a ginglymoid distal articulation. Remaining phalanges III-3, IV-1, IV-2, IV-3 and IV-4 have rounded distal articulations and concave proximal articulations with well-developed collateral ligament pits distally, at least on the lateral side, and shafts that arch slightly dorsally. Digit III is likely significantly longer than digit IV, in contrast to some other more derived troodontids [46], [81], [83].

### Histology

**Figure 7:**
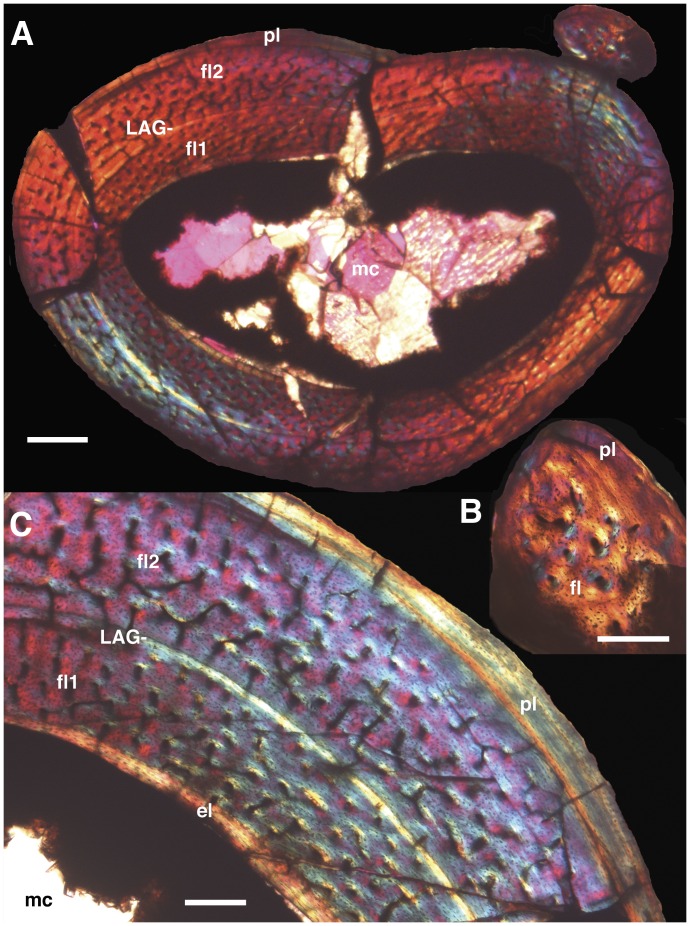
Histology of the right tibia and fibula of *Mei long*, DNHM D2514. **A**, cross-section of tibia and fibula in articulation. **B**, partial cross-section of fibula. **C**, partial cross-section of tibia. Note two zones of fibro-lamellar bone separated by a LAG in the tibia and the largely avascular lamellar bone that marks the periphery of both elements. The latter can be interpreted as an external fundamental system. Abbrevaitions: **el**, endosteal lamellar bone; **fl**, fibro-lamellar bone; **fl1**, first and interior zone of fibro-lamellar bone; **fl2**, second and more exterior zone of fibro-lamellar bone; **LAG**, line of arrested growth; **mc**, medullary cavity; **pl**, peripheral lamellar bone. Scale bars equal 0.5 mm in **A** and 0.2 mm in **B** and **C**.

We removed a mid-length section of the right tibia and fibula of DNHM D2154 for histologic sampling. The small size of the individual and its articulated nature allowed for the tibia and fibula to be thin-sectioned together (Figure 7). The fibula cross-section measures 0.86 by 0.63 mm and lacks an open medullary cavity (Fig. 7B). Instead, the cross-section possesses two distinct histologic regions: an interior, elliptical region of fibro-lamellar bone with approximately 20 longitudinally oriented primary osteons; and multiple exterior chevron-shaped zones with diffuse banding and only a few, more circumferentially arranged osteons. The latter zones narrow both anteriorly and posteriorly and show a parallel extinction pattern under crossed-polars indicative of lamellar bone.

The elliptical cross-section of the tibia measures 3.4 deep by 4.85 mm medio-laterally and has a straighter anterior face and a more convex posterior face (Fig. 7C). The well-developed medullary cavity mirrors the overall cross-section in shape. The cavity lacks any cross-cutting trabeculae. Permineralization consists of orange to opaque mineral growth along the interior of the cortical bone from which emerge small lath-like crystals. Sparry calcite fills the central and a majority portion of the medullary cavity.

Cortical bone thickness ranges from 0.54 to 0.93 mm. A thin, 0.001–0.05 mm thick band of circumferential lamellar bone, devoid of osteons marks the endosteal portion of the cortex. The orientation of the long axes of lacunae and of the crystals as visible under crossed polars concurs with a circumferential arrangement.

The majority of the cortex consists of two zones of fibrolamellar tissue separated by a band of circumferential and largely avascular bone with a single line of arrested growth (LAG) (Fig. 7). The interior zone of fibrolamellar bone is 0.32 to 0.50 mm thick and possesses longitudinally aligned osteons. These osteons are regularly spaced but do not form discrete circumferential bands. The band separating the two zones possesses circumferentially oriented lacunae and crystals. The latter are evidenced by parallel extinction patterns under crossed polars. This band also bears a distinct LAG visible through the entire circumference and only a few osteons occur in proximity to the LAG. The second, more external zone of fibrolamellar tissue is largely similar to the interior one and measures 0.30–0.50 mm in thickness. It differs in that not all the osteons are longitudinally oriented and instead include some circumferentially arranged osteons primarily close to the separating band and others radially oriented.

The periphery of the bone along the posterior aspect shows a decrease in osteon and vessel density vessels by as much as 0.2 mm. Additionally, there is a partial realignment of osteon orientation. In contrast, a circumferentially oriented band of avascular lamellar bone with circumferentially oriented lacunae and crystals comprises the remaining two-thirds of the periphery. This tissue reaches a maximum thickness of 0.15 mm on the anterior aspect of the cross-section and then tapers around the lateral and medial edges. LAGs or bands within this tissue are consequently not traceable through the entire bone’s circumference.

### Phylogenetic Analysis

A total of 21 characters had coding changes, most of these due to better preservation or exposure of several regions of the skeleton (dentary, metacarpals, proximal phalanges, ilium) in DMNH D2154 compared to the holotype. The specific coding changes to the matrix can be found in Table 1. Seventeen characters were changed from “?” to “0.” Characters 214, 229, and 337 were changed because the better preservation of the ilium of DMNH D2154 provided new information. Characters 224, 231, 253 and 261 were changed because the orientation of the skull in DMNH D2154 provides a different view of the dentary, displaying details that were more obscured in the holotype’s skull. Better exposure of the maxillary teeth permitted coding of character 259. Characters 284, 285, 288, 292, 293, 294, 302, 328 and 344 were recoded because the left manus of DMNH D2154 is more openly preserved than the mani of the holotype and permits all of the requisite measurements to be made. Two characters were changed from “?” to “1.” Character 226 was originally not coded because the cranial surfaces of the distal ends of both holotype humeri face into matrix or other skeletal elements. These areas are exposed in DMNH D2154. Character 303 was originally coded as “?” because of the aforementioned poor preservation of the holotype ilia.

**Table 1:** Character changes to the coding for *Mei long* from Xu et al. (2011).

Coding changed from “?” to “0″	Coding changed from “?” to “1″		Characters which are polymorphic for *Mei long*	
214	226	139		
224	303	199		
229				
231				
253				
259				
261				
284				
285				
288				
292				
293				
294				
302				
328				
337				
344				

Two characters were found to be polymorphic between the two specimens of *Me*i. Character 139, “Scapula longer than humerus (0) or humerus longer than scapula (1),” was coded as “1” for the holotype and “0” in the Dalian specimen. The proportions from the two specimens closely straddle the demarking line. Character 199, “Distal end of metatarsal III smooth, not ginglymoid (0) or with developed ginglymus (1),” was coded as “1” for the holotype and “0” for the Dalian specimen. The complete matrix can be found in the attached nexus file (Analysis S1). We follow Makovicky et al. [85] and Turner et al. [86] in treating *Nequenraptor argentinius* [87] as a junior synonym of *Unenlagia comahuensis* [88].

**Figure 8:**
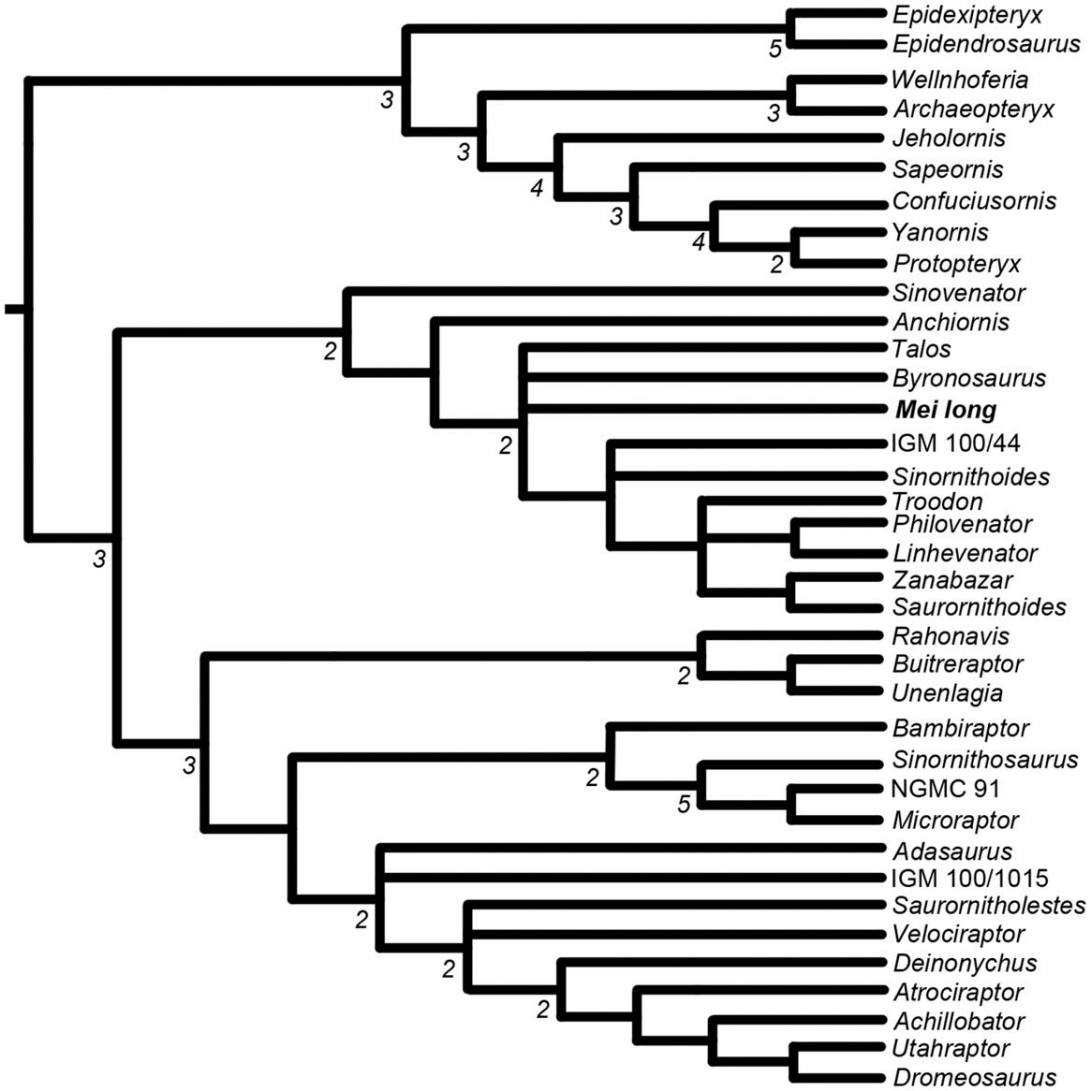
Phylogenetic relationships of *Mei long* within paravian portion of the fuller analysis of Coelurosauria. See Figure S1 for the remainder of the tree. Strict (Nelson’s) Consensus of 36 most parsimonious trees with a length of 1321 steps resulting from the analysis of the matrix from Xu et al. [24] with *Talos sampsoni* added. Numbers below branches represent Bremer decay indices greater than one. Protocol in text.

The analysis yielded 36 shortest trees with a length of 1,321 steps. The CI and RI of these trees is 0.336 and 0.743 respectively. The resulting strict consensus tree (Figure 8) is generally similar in overall topology of the clade Troodontidae to Figure 8 of Xu et al. [23]. The position of *Mei* is recovered as a basal troodontid (Figure 8), as occurs in Xu et al. [23] and is consistent with other recent analyses of the group [23]–[27]. *Mei* is in a polytomy with *Byronosaurus* and *Talos*. There is reduced resolution between *Sinornithoides* and IGM 100/44. The relationships among the more derived troodontids show an increased resolution, with *Philovenator* recovered as the sister taxon to *Linhevenator*, and *Zanabazar* and *Saurornithoides* being recovered in a small clade. The relationships between *Troodon* and these other parings remain unresolved. As mentioned by Zanno et al. [25], a complete reevaluation of North American troodontid specimens is necessary to improve resolution in the crown of Troodontidae.

*Talos* is recovered in a polytomy with *Mei*, *Byronosaurus*, and a clade including all more crownward troodontids. A previous analysis has recovered *Talos* closer to *Zanabazar*, *Troodon*, and *Saurornithoides* [25], though the same study also recovered *Talos* close to *Bryonosaurus* in one alternate configuration. It is interesting that these two genera are again closely recovered, especially since they have very little overlapping material [25], [42]. Given the incomplete nature of *Talos*, the lack of cranial material, and the coding of this taxon from the literature, we would strongly caution that this result is preliminary. We agree with others that more complete material for several troodontid species is necessary to better resolve troodontid phylogeny [25]. This analysis demonstrates that the new morphological findings from the Dalian specimen do not significantly alter the position of *Mei long*.

## Discussion

Using the diagnosis of Makovicky and Norell [49], several unambiguous characters diagnose specimen DNHM D2154 to Troodontidae. The labial face of the dentary bears a groove that contains exits for neurovascular foramina. The quadrate bears a pneumatic foramen on its caudal aspect. There are a large number of teeth and the teeth of the anterior dentary and maxilla are closely packed. The distal caudal vertebrae lack a neural spine but instead bear a sulcus on the dorsal midline.

Specimen DNHM D2154 is referable to *Mei long* [16] on the basis of sharing the following diagnostic traits: a troodontid possessing and extremely large nares extending caudally over one half of the maxillary tooth row [16]; closely packed middle maxillary teeth; maxillary tooth row extending caudally to the level of the preorbital bar [16]; and the most proximal end of the pubic shaft is significantly compressed craniocaudally and extends laterally just ventral to the articulation with the ilium [16].

The cervical vertebrae are similar in overall form to those known from other troodontids [52], [34], [40], [53]. The low neural spines and a ventral keel of DMNH D2154 are diagnostic for troodontids [48]. The specimen shares the following characteristics with *Saurornithoides mongoliensis* [55]: (1) the position of the parapophyses, (2) the concave cranial articular surface of the centrum, (3) the position of pneumatic openings, (4) long prezygapophyses that diverge from the midline, and (5) round and concave postzygapophyseal articular surface. As in *Sinornithoides* [36], the centra are parallelogram-shaped in lateral view (Fig. 3A), and the ventral surface of the centra are flat anteriorly and slightly keeled posteriorly.

Two features with potentially important functional implications are the lengthening of metacarpal III beyond that of II and the broad and posterioly expanded pelvis. An elongate digit III has been linked with arboreality [72]. Although some posterior flaring may occur in the pelvis of dromaeosaurids including *Microraptor* [60], [89], the sacrum of *Mei long* is noteworthy for its breadth, particularly in one of the smallest troodontids.

Despite the wealth of anatomical detail preserved in this nearly complete specimen, a recoding of the species for the characters in the phylogenetic matrix of Xu et al. [23] yielded few changes in its position in Troodontidae. The most significant changes were a small loss of resolution between *Sinornithoides* and IGM 100/44, and a loss of resolution around *Mei* relating to the position of *Talos* and *Byronosaurus*. The relative position of *Mei long* is not significantly affected, however.

### Maturity

Although DNHM D2154 exhibits several juvenile attributes, the degree of axial fusion, limb proportions, bone surface texture, and bone histology argue for a mature individual. Several of the features of the vertebral column indicate the specimen is somatically mature. As assessed on cervicals 4 and 6, the cervical arches and centra are fully fused. The dorsal arches also appear to be fully fused to the centra. The arches and spines of the sacral vertebrae are fused into a single neural platform bearing one blade-like spine. All of the caudal neural arches that are visible are fully fused. The fusion of neural arches to centra has been shown to be a good proxy of maturity in crocodyliform archosaurs [90] and suggested to be useful in theropod dinosaurs [91]. Brochu [90] observed a sequence of fusion beginning with the caudal vertebrae and progressing cranially with increasing maturity. In *Troodon formosus* specimens from Montana, fusion of vertebrae to form the sacrum occurs after neurocentral fusion is completed throughout the vertebral column and in a presumably reproductively active adult (MOR 748) associated with eggs [92]. That all of the observed neural arches, including those of the cervical series as well as the sacrum, are fused in DNHM D2154, suggests that the animal was sexually mature and approaching full maturity at the time of death. This conclusion is further supported by the bone surface texture.

The surfaces of the long bones are smooth and the bone appears dense. This is in contrast to the porous texture noted in embryonic bird bones [93] and the grooved textures seen in more mature, but still young birds [94]. These observations are deemed relevant in light of the theropod ancestry of birds [95]–[98].

Several histologic features argue that DNHM D2154 represents a mature individual probably more than two years old. First, the periphery of both elements lacks any developing primary osteons. Instead, circumferentially oriented lamellar tissue comprises the bulk of the exterior bone. The thickness of this slow-growing tissue along the cranial aspect of the tibia far exceeds that separating the two zones of fibrolamellar tissue, suggesting a longer and more protracted period of slow growth than that associated with the preserved LAG. This peripheral tissue has the appearance of an external fundamental system, and its presence is consistent with the finished appearance of bone surfaces throughout the skeleton noted above. Additionally, the presence of inwardly growing endosteal lamellar bone in the tibia indicates that the medullary cavity was not expanding. Consequently, growth appears to have been minimal at the time of death in this individual. The two similarly sized zones of fibro-lamellar tissue separated by a LAG in the tibia show that earlier and otherwise rapid growth had experienced a single slowdown. Because earlier expansion of the medullary cavity may have erased earlier LAGs, we retrocalculated the presence of LAGs using the thickness of the second zone of fibro-lamellar bone and the bone diameter represented by the preserved LAG. Potentially, remodeling of the relatively large medullary cavity of the tibia could have removed one or possibly two earlier LAGs. Thus, interpreting the preserved LAG and the exterior lamellar bone as annual in origin would imply an age of minimally over two and possibly over four years old.

In contrast to the above, a few features of the specimen are typically associated with immaturity. The cervical ribs are unfused. The paired frontals and possibly the nasals are not fused. While typically indicating immaturity [99], unfused frontals are also seen in some large individuals of *Troodon*, e.g. MOR 553 [25], indicating that they may not be good indicators of immaturity in troodontids. The shape of the skull may also be typical of immature individuals [34], [94], [100]. The type of *Mei long* also exhibits unfused nasals and frontals, and has a skull with a steep rostral profile [16]. One possible conclusion is that *Mei long* is a paedomorphically small troodontid and these features are typically juvenile theropod features retained into adulthood. This might explain the juxtaposition of juvenile and adult features seen in these specimens.

This *Mei long* specimen is similar in size to *Anchiornis*, with a slightly longer femur but shorter tibiotarsus and metatarsals. The equation of Anderson et al. [101] estimates a mass for DNHM D2154 of approximately 420 g. Thus, *Mei long*, including the type, is one of the smallest species among non-avian dinosaurs [86].

Turner et al. [86] argued that small body size was primitive for all Paraves, as well as both the Troodontidae and Dromaeaosauridae. Additional description and phylogenetic reconsideration of a second *Mei long* specimen, which is mature and relatively basally positioned within Troodontidae, lends further support to the Turner et al. [86] hypothesis.

As evidenced by *Mei long*, the size reduction of paravians relative to more primitive theropods was apparently not accomplished by a major change in growth. *Mei long*, despite a body size orders of magnitude smaller, exhibits multi-year growth and cortical bone with zones of fibro-lamellar bone separated by LAGs as found in more basal theropods like *Megapnosaurus* (formerly *Syntarsus*), *Allosaurus*, and tyrannosaurids [102]–[105]. This primitive growth pattern was then maintained through troodontids [25], [93], [102], [106], [107].

### Behavioral and Taphonomic Implications

A major portion of the original description of *Mei long* involves discussion of the significance of the articulated position of the skeleton and the mode of preservation of the specimen [16]. Subsequent studies have provided alternative processes that could have produced the posture of the specimen, requiring a more detailed treatment of the position of the specimen [13], [15]. Specimen DNHM D2154 retains a nearly identical posture in comparison to the type specimen of *Mei long*. In both, the tail curls forward and under the neck, hind and fore limbs lie folded beneath the body, and the neck and head curve back between the shoulder and folded elbow toward the hindlimb (Fig. 1). They differ primarily in that in DNHM D2154 the neck arches to the left rather than to the right as in the holotype [16].

The position of these *Mei long* specimens also matches that of the holotype of *Sinornithoides* [36], [46]. While much of the dorsal half of that specimen had been eroded before discovery, what remains is consistent with the specimens of *Mei long*. The legs are folded and the tail is curled in front of the feet. Further, the skull is preserved behind the left elbow, above the manus. The arms are folded at the sides, with the elbows pointing laterally to dorsolaterallym and the manual digits are folded under the body towards the feet, with digit I closest to the hypothesized ground. The *Sinorntihoides* specimen comes from a sandstone of the largely aeolian Eiginhelue (Yiginholo) Formation of the Ordos Basin, Inner Mongolia, China [36], [108].

The presence of three specimens representing two troodontid species in nearly identical positions suggests that the position of these animals is not random. Xu and Norell [16] interpreted this posture as a stereotypical brooding or sleeping position and suggest three implications. First, preservation occurred in such a manner that burial retained the life posture of the animal as it lay resting on the ground. This may have resulted from Pompei-like deposition of tuffaceous ashes. Further, this posture represents a behavior shared by theropods such as troodontids and perhaps oviraptorids with modern birds. Finally, this posture may have served to reduce surface area and conserve heat for a homeothermic physiology [16].

The preserved postures of these troodontids differ markedly from some perimortem postures resulting from a variety of causes of death. An opisthotonic posture consists of hyperextension of the spine with both neck and tail curving over the back. According to one proposal, this posture represents death throes and a response of the central nervous system to various afflictions such as asphyxiation, toxins, or infections [109] (but see an opposing proposal in [110]). The inferred cause of death for the Jehol Biota in plinean ash-fall scenarios is often asphyxiation due to exposure to toxic volcanic gasses or ash [111]. The troodontid specimens also lack the “pugilistic” hyperflexion of hands and toes as commonly seen in humans that perished in pyroclastic flows and surges at Herculaneum and Pompei, Italy, a known perimortem response to death from high temperatures and fire [112], [113]. However life- and sleep-like postures are retained in a number of the Pompei victims [113].

Absence of opisthotonic or pugilistic postures in these troodontid specimens likely has implications for their taphonomic history. These might include: (1) burial occurring faster that perimortem response; (2) death occurring by a mechanism incapable of stimulating a perimortem posture; or (3) an absence of such responses in troodontids. The last seems unlikely, given that a number of theropod specimens preserve opisthotonic postures [109], [114]. Presence of life postures in these three troodontid specimens constrains reconstructions of their taphonomic history and contradicts burial in a low energy lahar, as has been proposed for other well-preserved specimens from the lower Yixian Formation of Liaoning [7]. Even a low energy lahar is likely to move a small animal out of a perimortem position.

Exposure must have been sufficiently limited either temporally or spatially to prohibit significant decay or scavenger activity. We suggest that sheltering within a burrow might be one possible mechanism to explain these specimens. Alternatively, the postures in *Mei long* and *Sinornithoides* may reflect protective responses to volcanic and eolian events, respectively. Unfortunately, like many fossils from the Yixian Formation, detailed stratigraphic and taphonomic data for the *Mei long* specimens are lacking, and only limited taphonomic information has been published for *Sinornithoides*. Consequently, many questions remain about their preservation.

## Supporting Information

Figure S1 The non-paravian portion of the phylogenetic analysis of Coelurosauria described in the text. Strict (Nelson’s) Consensus of 36 most parsimonious trees with a length of 1321 steps resulting from the analysis of the matrix from Xu et al. [24] with *Talos sampsoni* added. Numbers below branches represent Bremer decay indices greater than one. Protocol in text.
(TIF)

Table S1 Selected measurements of *Mei long* specimens in millimeters. R and L indicate right and left elements, when applicable. *denotes estimated lengths.
(DOC)

Analysis S1 A nexus file containing the matrix used to produce the trees seen in Figures 8 and S1. This file is formatted for use with the program TNT.
(NEX)
