# Supplementary figures and images for: A Second Soundly Sleeping Dragon: New Anatomical Details of the Chinese Troodontid Mei long with Implications for Phylogeny and Taphonomy
Source: PLoS One. 2012 Sep 27;7(9):e45203. doi: 10.1371/journal.pone.0045203 (PMC3459897; doi:10.1371/journal.pone.0045203)

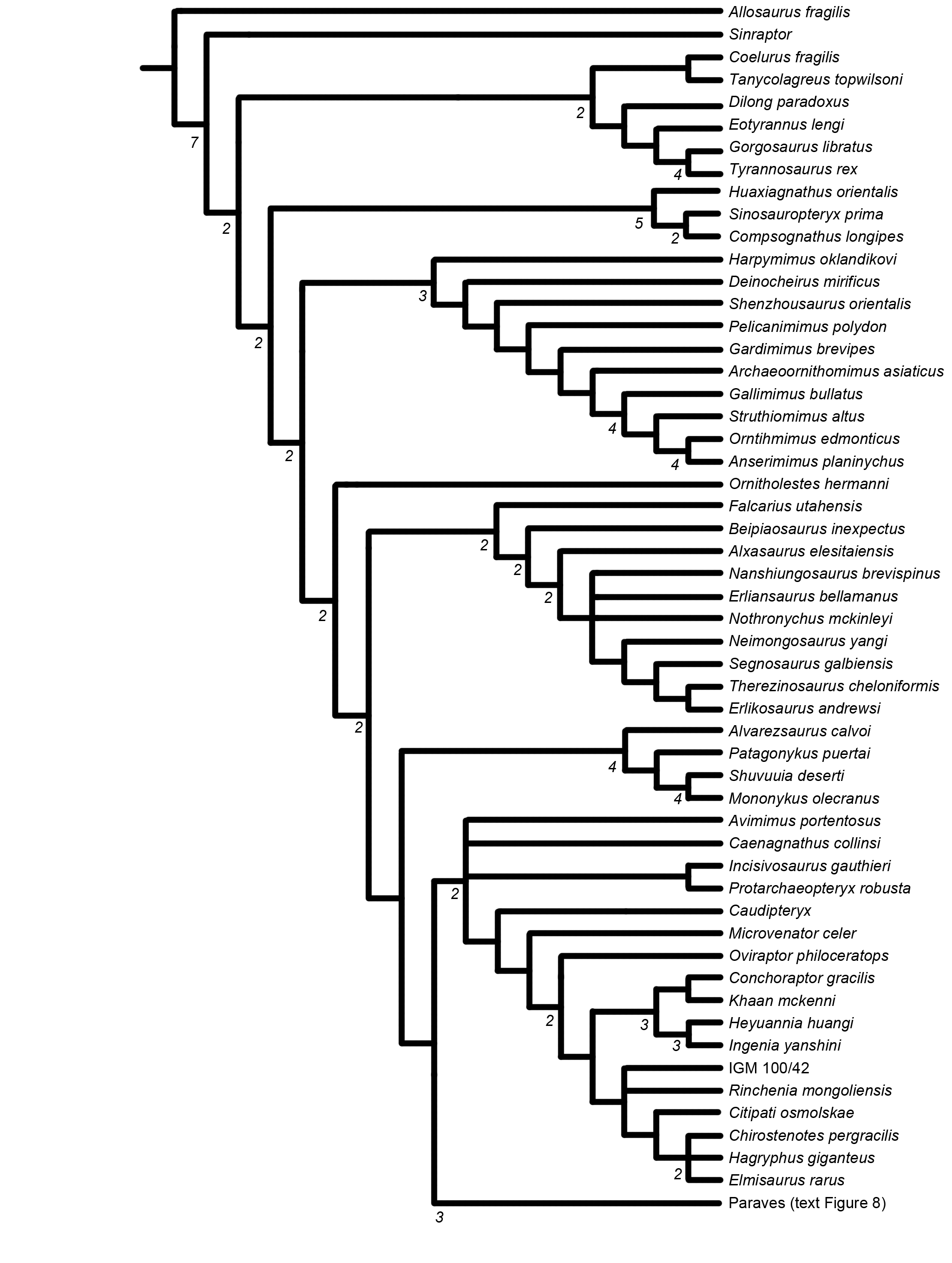

Supplement: Figure S1 — The non-paravian portion of the phylogenetic analysis of Coelurosauria described in the text. Strict (Nelson’s) Consensus of 36 most parsimonious trees with a length of 1321 steps resulting from the analysis of the matrix from Xu et al. [24] with Talos sampsoni added. Numbers below branches represent Bremer decay indices greater than one. Protocol in text. (TIF) [file pone.0045203.s001.tif]
