# Supplementary material for: A Second Soundly Sleeping Dragon: New Anatomical Details of the Chinese Troodontid Mei long with Implications for Phylogeny and Taphonomy
Source: PLoS One. 2012 Sep 27;7(9):e45203. doi: 10.1371/journal.pone.0045203 (PMC3459897; doi:10.1371/journal.pone.0045203)
Supplement: Table S1 — Selected measurements of Mei long specimens in millimeters. R and L indicate right and left elements, when applicable. *denotes estimated lengths. (DOC) [file pone.0045203.s002.doc]

TABLE 1. Selected measurements of *Mei long* specimens in millimeters. R and L indicate right and left elements, when applicable. *denotes estimated

|  | | holotype (IVPP V12733) | | Referred specimen (DNHM D2154) | |
| --- | --- | --- | --- | --- | --- |
| Skull, length | 53 | | *49.0 | |  |
| Skull, width across frontals |  | | 17.5 | |  |
| Skull, greatest height |  | | *21.0 | |  |
| Maxilla, length |  | | 16.5 (L) | |  |
| Centrum length, D9 |  | | 5.8 | |  |
| Centrum length, D10 |  | | 6.0 | |  |
| Centrum length, D11 |  | | 5.9 | |  |
| Centrum length, D12 |  | | 5.9 | |  |
| Sacrum, total length | 30 | | 22.2 | |  |
| Sacrum, width at S1 |  | | *14.3 | |  |
| Sacrum, width at S2 |  | | *14.3 | |  |
| Sacrum, width at S3 |  | | *19.3 | |  |
| Sacrum, width at S4 |  | | 24.7 | |  |
| Sacrum, width at S5 |  | | 24.4 | |  |
| Centrum length, Caudal (C)4 |  | | 4.0 | |  |
| Centrum length, C5 |  | | 4.0 | |  |
| Centrum length, C6 |  | | 4.3 | |  |
| Centrum length, C7 |  | | 4.9 | |  |
| Centrum length C8 |  | | 5.5 | |  |
| Centrum length C9 |  | | 8.0 | |  |

TABLE 1 (Continued)

| Centrum length C10 |  | 10.3 |
| --- | --- | --- |
| Centrum length C11 |  | 11.1 |
| Centrum length C12 |  | 11.0 |
| Centrum length C13 |  | 11.2 |
| Centrum length C14 |  | 10.9 |
| Centrum length C15 |  | 10.9 |
| Centrum length C16 |  | 10.6 |
| Centrum length C17 |  | 10.6 |
| Scapula, total length | 45 | 36 (L), 40 (R) |
| Scapula, caudal width |  | 5 (R) |
| Scapula, width midshaft |  | 2.5 (L), 2.5 (R) |
| Scapula, cranial width |  | 5.2 (L) |
| Humerus, total length | 42 | 36 (L) |
| Humerus, craniocaudal midshaft thickness |  | 2.5 (L) |
| Humerus, mediolateral midshaft thickness |  | 2.5 (L) |
| Humerus, craniocaudal width distal end |  | 6.7 (L) |
| Humerus, mediolateral width distal end |  | 3.5 (L) |
| Ulna, length |  | *34 (L) |
| Metacarpal I, total length |  | 4.0 (R) |
| Metacarpal II, total length |  | 13.0 (R) |
| Metacarpal III, total length |  | 14.3 (R) |

TABLE 1 (Continued)

| Maunal phalax length I-1 |  | 14.0 (R) |
| --- | --- | --- |
| II-1 |  | 7.8 (R) |
| II-2 |  | 13.0 (R) |
| III-1 |  | 8.1 (R) |
| III-2 |  | 8.1 (R) |
| III-3 |  | 10.7 (R) |
| Ilium, total length |  | 34.4 (R) |
| Preacetabular blade, max depth |  | 8.3 (R) |
| Ilium depth at pubic pedicel |  | 12.3 (R) |
| Ilium depth at ischial pedicel |  | 7.7 (R) |
| Length of acetabulum (interior) |  | 7.3 (R) |
| Total femur length | 81 | *65 |
| Tibiotarsus total length | 106 | *86.1 |
| Metatarsus total length |  | *49.0 |
| Metatarsal II, length |  | 42.5 |
| Metatarsal III, length | 58 | *49 |
| Metatarsal IV, length |  | 45.7 |
| Metatarsal V, length |  | 11.0 |
| Pedal phalanges length III-1 |  | 12.8 |
| III-3 |  | 7.6 |
| IV-1 |  | 8.4 |
| IV-2 |  | 6.4 |
| TABLE 1 (Continued) |  |  |
| IV-3 |  | 5.9 |
| IV-4 |  | 5.2 |
